# Creutzfeldt-Jakob disease: Importance of early magnetic resonance imaging

**DOI:** 10.4103/0972-2327.48859

**Published:** 2009

**Authors:** K. S. Madhusudhan, Chandan Jyoti Das

**Affiliations:** Department of Radiodiagnosis, All India Institute of Medical Sciences, New Delhi – 110 029, India

**Figure 1:**
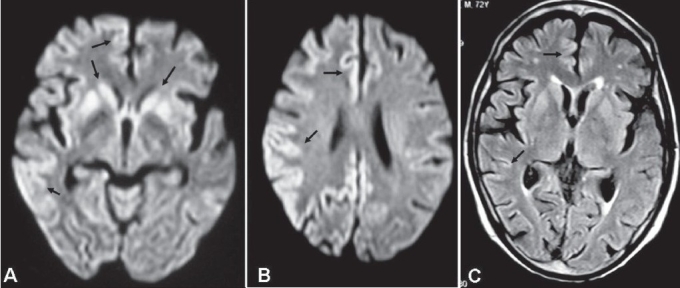
Diffusion weighted MRI sequence (A and B) shows symmetrical hyperintensity involving bilateral caudate nucleus, globus pallidus, putamen, and cerebral cortex (arrows). The FLAIR image (C) also shows diffuse hyperintensity involving the cerebral cortices and basal ganglia (arrows)

A 72-year-old male presented with ten days history of altered sensorium, involuntary movement of limbs, and progressive dementia. There was no history of fever or vomiting. On examination, the patient was disoriented, the muscle tone was increased, and tendon reflexes were exaggerated. The Babinski's sign was positive bilaterally. There were no signs of meningeal irritation or cerebellar signs. The EEG was normal. MRI done as a part of routine checkup showed diffuse hyperintensity in the cortex of bilateral frontal and parietal lobes and in bilateral caudate nuclei, globus pallidi, and putamina on diffusion weighted [DWI; Figure 1A and Figure 1B] and fluid-attenuated inversion recovery (FLAIR) images [Figure 1C] with restriction of diffusion. Imaging appearance was typical for CJD in the clinical setting. Subsequent CSF analysis for 14-3-3 protein was positive, confirming the diagnosis.

CJD is a transmissible, rapidly progressive, invariably fatal neurodegenerative disorder.[1] It is caused by the accumulation of abnormal prion protein in the neurons resulting in their spongiform degeneration.[1] Clinical diagnosis is often difficult and needs exclusion of other diseases producing similar symptoms. MRI, especially the DWI sequence, plays a vital role in suggesting an early diagnosis. It characteristically shows symmetrical T2 hyperintensities with restricted diffusion in bilateral cerebral cortices and basal ganglia.[2,3] Confirmation of the disease requires demonstration of periodic sharp wave complexes on EEG or 14-3-3 protein in CSF.[4] The disease is transmissible and early diagnosis is helpful in appropriate patient management. The disease is fatal and no treatment is currently available.
